# Radiotherapy resistance acquisition in Glioblastoma. Role of *SOCS1* and *SOCS3*

**DOI:** 10.1371/journal.pone.0212581

**Published:** 2019-02-27

**Authors:** Maria Paz Ventero, Maria Fuentes-Baile, Cristina Quereda, Elizabeth Perez-Valeciano, Cristina Alenda, Pilar Garcia-Morales, Danilo Esposito, Pilar Dorado, Victor Manuel Barbera, Miguel Saceda

**Affiliations:** 1 Hospital General Universitario de Elche, FISABIO, Camí de l'Almazara, Elx (Alicante), Spain; 2 Instituto de Investigación Biomédica y Sanitaria de Alicante (ISABIAL), Hospital General Universitario de Alicante, C/ Maestro Alonso, Alicante (Alicante), Spain; 3 Departamento de Fisiología, Genética y Microbiología, Facultad de Ciencias, Universidad de Alicante (Alicante), Spain; 4 Instituto de Biología Molecular y Celular, Ed. Torregaitan, Universidad Miguel Hernández, Elche (Alicante), Spain; 5 Unidad de Oncología Radioterápica, ERESA, Hospital General Universitario de Elche, Camí de l'Almazara, Elx (Alicante), Spain; Universidad de Castilla-La Mancha, SPAIN

## Abstract

Glioblastoma multiforme (GBM) is a poor prognosis type of tumour due to its resistance to chemo and radiotherapy. *SOCS1* and *SOCS3* have been associated with tumour progression and response to treatments in different kinds of cancers, including GBM. In this study, cell lines of *IDH*-wildtype GBM from primary cultures were obtained, and the role of *SOCS1* and *SOCS3* in the radiotherapy response was analysed. Fifty-two brain aspirates from GBM patients were processed, and six new cell lines of *IDH*-wildtype GBM were established. These new cell lines were characterized according to the WHO classification of CNS tumours. *SOCS1* and *SOCS3* expression levels were determined, at mRNA level by Q-PCR, at protein level by immunocytochemistry, and Western blot analysis. The results showed that *SOCS1* and *SOCS3* are overexpressed in GBM, as compared to a non-tumoral brain RNA pool. *SOCS1* and *SOCS3* expression were reduced by siRNA, and it was found that *SOCS3* inhibition increases radioresistance in GBM cell lines, suggesting a key role of *SOCS3* in radioresistant acquisition. In addition, radioresistant clonal populations obtained by selective pressure on these cell cultures also showed a significant decrease in *SOCS3* expression, while *SOCS1* remained unchanged. Furthermore, the induction of *SOCS3* expression, under a heterologous promoter, in a radiotherapy resistant GBM cell line increased its radiosensitivity, supporting an important implication of *SOCS3* in radiotherapy resistance acquisition. Finally, the treatment with TSA in the most radioresistant established cell line produced an increase in the effect of radiotherapy, that correlated with an increase in the expression of *SOCS3*. These effects of TSA disappeared if the increase in the expression of *SOCS3* prevented with an siRNA against *SOCS3*. Thus, *SOCS3* signal transduction pathway (JAK/STAT) could be useful to unmask new putative targets to improve radiotherapy response in GBM.

## Introduction

Glioblastoma multiforme (GBM) is the most common malignant tumour of the central nervous system (CNS) in the adult population, its incidence being around 2–3 people per 100.000 in USA and Europe. It is considered a very aggressive and lethal tumour, because there is not an effective therapy to date, thereby, being an incurable type of cancer [1,2].

GBM is usually divided in two groups, depending on their origin: primary GBM’s which are developed “*de novo*”, and low-grade gliomas that become GBM, denominated secondary GBM. Both are histologically identical, but primary GBM has worse prognostic than secondary GBM [3,4].

Recently, the World Human Organization (WHO) has updated the classification of CNS tumours, and has included molecular marks, which enable to identify the gliomas, and also distinguish between primary and secondary GBM. This classification has established *IDH*-wildtype GBM and *IDH*-mutant GBM; *IDH*-wildtype GBM has a worse prognosis, with a survival rate around 10 to 15 months, it often has mutations in the *TERT* promoter (76%), and sometimes harbours alterations in *TP53* (27%) and *PTEN* (24%). *IDH*-mutant GBM has a better prognosis and the survival rate is increased to 31 months. In this type of tumours, the mutations of *TERT* promoter are rare (26%) and *TP53* and *PTEN* alterations are also present in a low percentage [5].

The current therapy for GBM includes resection surgery, followed by radio and chemotherapy, frequently given together to obtain a synergistic effect. The radiotherapy treatment consists of five consecutive sessions of 2Gy at a 6Gy/min dose rate per week for six weeks, the total treatment being 60Gy [6]. The most common drug used in chemotherapy in GBM is Temozolomide (TMZ), which is administered concomitant with radiotherapy [7]. Additionally, sometimes, during resection surgery, wafers impregnated with Carmustine (BCNU) are implanted in the environment of the tumour [8].

Despite of these aggressive treatments, the survival rate is only increased in a few months, because GBM has different ways to acquire resistance to chemo and radiotherapy, either activating DNA repair system or producing alterations in the cell cycle and apoptosis regulation [9,10]. Resistance to chemotherapy has been extensively studied, and it is mainly due to *MGMT* (0–6 methylguanine-DNA Methyltransferase) gene expression. MGTM is a protein involved in the DNA repair system, which is able to avoid DNA damage caused by TMZ or BCNU [11,12]. However, there is no much knowledge about the mechanisms related to radiotherapy resistance in GBM, and the basic mechanism of its acquisition remains unclear.

SOCS1 and SOCS3 proteins are members of the Suppressors of Cytokine Signalling (SOCS) family. Both are implicated in the signal regulation of JAK/STAT pathway, which is involved in cell proliferation and apoptosis [13]. Commonly, the constitutive activation of this pathway has been considered a hallmark of several cancers [14,15]. On the other hand, SOCS proteins alterations have been associated to different diseases, including cancer [16]. In this sense, the methylation status of *SOCS3* has been proposed as a malignant prognostic biomarker [17], and the differential expression of *SOCS1* and *SOCS3* in GBM has been studied as putative factors involved in radiotherapy resistance [18]. Likewise, the expression of these genes has been related with radiotherapy response in other types of cancer, such as a gastric or cervix cancer [19,20].

On the other hand, histone deacetylases inhibitors (iHDACs) have been proposed as new anti-cancer agents [21], due to their capability to decrease the tumour progression and to increase the radio and chemosensitivity in different tumour cell lines [22–24]. Moreover, the iHDACS have been related to JAK-STAT pathway and *SOCS1* and *SOCS3* expression [25].

In this study, six human cell lines of *IDH*-wildtype GBM, established from primary cultures of GBM patients, have been obtained and characterized according to the current WHO classification. In addition, they have been tested for their chemo and radiotherapy sensitivity. The results showed that *SOCS1* and *SOCS3* are overexpressed in primary GBM, and also demonstrate a relationship between *SOCS3* expression and radiotherapy resistance acquisition, suggesting the utility of *SOCS3* and its signal transduction pathway as a new source of therapeutic targets.

## Materials and methods

### Cell culture

GBM primary cell cultures were obtained from brain aspirates of patients who have been diagnosed with GBM. These brain aspirates came from resection surgery of patients older than 18 years old, which had signed the informed consent. The procedures for obtaining the tissue samples were developed in accordance with the national ethical and legal standards, and following the guidelines established in the Declaration of Helsinki (2000). The research project was conducted under the written approval of the Ethic Committee of Clinical Research (CEIC) of the “Hospital General Universitario de Elche (Spain)”, and in collaboration with the Hospital Biobank, which are included in the Valencian Network of Biobanks.

The brain aspirates were centrifuged at 1.000 rpm, 10 min. The pellets obtained were resuspended in 3 volumes of Erythrocyte Lysis buffer (Qiagen), and incubated for 30 min, at 4°C. Then, they were again centrifuged in the same conditions, and the final pellets were resuspended in 10 ml of Dulbecco’s modified Eagle’s medium: Nutrient Mixture F-12 (Gibco) containing 10% Fetal Bovine Serum, qualified, heat inactivated (Gibco), and 1% of streptomycin and penicillium mixture (Biowest). This media was denominated completed DMEM F12. These cellular suspensions were sieved using the Sieve tissue grinder kit (Sigma Aldrich). The sieved solution was centrifuged at 1.000 rpm, 5 min. Then, it was performed an enzymatic digestion with hyaluronidase (4.5 U/ml), and collagenase II (0.09 mg/ml) for 30 min, at 37°C. Finally, cellular suspensions were centrifuged at 1.000 rpm, 5 min and resuspended with complete DMEM F12 media. The cell suspension was placed in a T25 flask and kept into an incubator with a controlled atmosphere (37°C, high humidity and 5% CO_2_). Maintenance of cell cultures consisted in regular passes, and media replacement until they reach a growth rate similar to cell lines.

### Immunocytochemistry of GFAP, SOCS1 and SOCS3

Cells were plated in a 24 well plates at a density of 75.000 cells per well, and kept for 24 hours into an incubator with controlled atmosphere. Then, cells were fixed with 4% paraformaldehyde in Dulbecco's Phosphate-Buffered Saline (DPBS) (Gibco) during 10 min, and they were permeabilized using 0.2% TRITON X-100 in DPBS for 10 min. In order to avoid unspecific unions blocking was carried out with a solution of 1% Bovine Serum Albumin in DPBS for 1 hour. At that point, cells were incubated with antibodies: anti-GFAP (AB5804, Millipore), anti-SOCS1 (ab9870, Abcam), or anti-SOCS3 (ab6030, Abcam) into a humidify chamber overnight, at 4°C. Primary antibodies were used to a 1/250 dilution. Next day, cells were incubated with a secondary antibody (Molecular Probes) used to a 1/1.000 dilution, and with DAPI for 2 hours, at room temperature. In parallel, the same protocol was performed for negative controls, but in this case, these cells were incubated only with the secondary antibody, without primary antibody. The immunostained cells were visualized by a Leica TCS SP2 confocal microscope (Leica Microsystems).

### Immunocytochemistry of P53 and PTEN

Cell lines were fixed using neutral formalin and included into paraffin to build TMA (Tissue Microarrays) sections. Then, sections were deparaffined, rehydrated and incubated with primary antibodies anti-P53 (DO-7, Dako) or anti-PTEN (6H2.1, Dako). Subsequently, the immunocytochemistry assay was performed with streptavidin-biotin technique, using the Immunostainer Techmate 500 (Dako), and the results were visualized by Envision System (DakoCytomation).

### Isolation of nucleic acids

DNA isolation was performed from frozen dry pellets of each cell line, using the DNAeasy Blood & Tissue kit (Qiagen). To carry out the ARN isolation, cells were stored in RNAeasy Lysis Buffer (Qiagen) plus 1% β-Mercaptoethanol (Sigma Aldrich), then the kit RNAeasy Plus (Qiagen) was used to isolate RNA, according to the manufacturer’s instructions.

### Western blot analysis

Protein extracts were obtained and Western blot analysis was performed following protocols previously describe [26]. For this analysis, 100 μg of proteins per lane were resolved on 5–20% polyacrylamide-gradient gels. SOCS3 protein was detected using a 1:500 dilution of the same antibody used in the immunocytochemistry studies mentioned above (ab6030, Abcam). GAPDH, was detected using a 1:5.000 dilution of a specific antibody against to this protein (60004-1-IG. Proteintech). The secondary antibody used was a 1:10.000 dilution of a peroxidase-conjugated anti-immunoglobulin (anti-IgG). For this study, three protein extracts pool from non-tumural tissue (P1234051, BioChain; NB820-59177, Novus Biologicals; 635318, Takara) were used as a control.

### Molecular characterization of *IDH1*, *IDH2* and *TERT*

The single nucleotide polymorphism of *IDH1*, *IDH2* and *TERT* genes were detected by PCR, following the protocol previously described [27,28]. In all cases, PCR was performed using 250 ng of DNA with AmpliTaq Gold 360 Master Mix (ThermoFisher Scientific). PCR products were resolved by capillary electrophoresis in the ABI-PRISM 3500 Genetic Analyzer (Applied Biosystem). Electropherograms were analysed by Sequencing Analysis V 5.4 Software (Applied Biosystem).

### Methylation status of *MGMT*

The methylation status of *MGMT* promotor in the cell lines was performed using SALSA MS-MLPA ME011.B1 Mismatch Repair Genes kit (MRC-Holland) according to the protocol provided by the manufacturer. The quantity of DNA used was 200 ng, the PCR products were resolved in ABI-PRISM 3500 Genetic analyser (Applied Biosystem). The data from the electrophoretic separation were analysed using GeneScan Software (Applied Biosystems), and Microsoft Excel spreadsheet templates provided by the manufacture. To determine the methylation status, the criteria proposed by Jeuken *et al* [29] have been used.

### Expression studies

Retrotranscription of RNA to cDNA was made with 1 μg of RNA, using the High-Capacity cDNA Reverse Transcription kit (ThermoFisher Scientific), according to the protocol provided by manufacturer.

Q-PCR was carried out in 96 wells plates, with a master mix containing 1μl of specific gene assay: *MGMT* (Hs01037698_M1), *SOCS1* (Hs00705164_s1) or *SOCS3* (Hs02330328_s1), 10 μl TaqMan Gene Expression Master Mix (all of them from ThermoFisher Scientific) and 4 μl of cDNA obtained by retrotranscription. Endogenous control used was *GAPDH*-VIC (ThermoFisher Scientific). As a reference sample, an RNA pool from normal brain (temporal lobe) tissue from 5 human adults was used. (BioChain). For all studies, 3 technical replicates were performed, and negative controls were carried out with water. Q-PCR assays were performed in the 7.300 Real Time PCR System (Applied Biosystems).

### Chemo and radiotherapy

The drug used for chemotherapy was BCNU. Cells were plated, treated with 50μM BCNU for 24 hours and harvested for cellular viabilities studies.

Radiotherapy was performed in the Radiotherapy Oncology Unit of the “Hospital General Universitario de Elche”, using a VARIANT 2100C linear accelerator. The dose was 7Gy, with a dose rate of 6Gy/min.

For both assays, cells were plated in 6 well plates and kept into an incubator with controlled atmosphere, for 24 hours. The radio or chemotherapy treatment was supplied, and cells were incubated again in the same conditions for an additional period of 24 hours.

To assess the drug effect, viability studies were performed using the Muse Count & Viability Assay Kit; radiation effect was determined by Muse Cell Cycle Assay Kit (MERK). The results were visualized by a MUSE Cell Analyzer (MERK).

### Obtain of clonal radioresistant populations

The GB39 and G242 cell lines were plated in 100 mm cell culture plates. Weekly, they were irradiated with a 5Gy dose until reach an accumulated dose of 45Gy. Only few isolated cells survived to this process. Each isolated cell was recovered using the Cloning cylinders glass (Sigma Aldrich) and plated in a 96 wells plate. When these cells started to proliferate, they were transferred to a 24 wells plate, and then to a 6 wells plates. Finally, clonal radioresistant populations were obtained from the isolated cells.

### Small interfering RNA assay

*SOCS1* and *SOCS3* expression inhibition was carried out by transfection with specific small interfering RNA (siRNA). The transfection was made with specific siRNA of *SOCS1* (siRNA.SOCS1, Invitrogen) and *SOCS3* (siRNA.SOCS3, Invitrogen) to a 10 μM concentration, mixed with 5 μl of Lipofectamine RNAiMAX (Invitrogen) diluted in OPTI-MEN (Gibco). As a negative control, the same solution was used with non-specific siRNA (siRNA.NS, Invitrogen). Transfected cells lines were maintained in this solution plus complete DMEM F12 media without antibiotic for 48 hours at 37°C in a humified 5% CO_2_/air atmosphere.

### Upregulation of *SOCS3* mRNA expression

In order to upregulated *SOCS3* expression, we used a synthetic plasmid which had the *SOCS3* Open Reading Frame (ORF) and a resistance cassette of Hygromycin under a heterologous promoter provided by Sinobiological (HG11315-UT). Transfection with a control plasmid was also performed to assess whether the transfection has an intrinsic effect. Cells were transfected with 15 ng or 25 ng of the plasmid using Lipofectamine 2.000 (Invitrogen) diluted in OPTI-MEN (Gibco) and grown in culture media plus Hygromycin to select only the transfected cells. Then, the culture was divided into 3 aliquots, one for the *SOCS3* expression studies, and the other two for cell cycle distribution studies one of them was irradiated, while the other was not.

To induce the increase in the expression of the endogenous levels of *SOCS3*, the cells were pre-treated for 16 h with different doses of TSA (25–200 nM) before proceeding to irradiation.

### Statistical analysis

Mean and SD values were calculated for *SOCS1* and *SOCS3* expression and cell cycle distribution data. Normal distribution of the data was tested by Shapiro-Wilk test and homoscedasticity was tested by Levene Test. Data from cell cycle distribution and percentages of SubG1/G2M showed a non-normal distribution. Data from expression studies showed a normal distribution, and homogeneity of variances. Parametric and non-parametric comparation test were used as required.

To paired comparisons between irradiated and non-irradiated phases of cell cycle, and between the percentages of SubG1/G2M of irradiated and non-irradiated cells Mann Whitney test was performed.

In the expression analysis, the followed criteria to consider alterations of expression were the proposed by Schmittgen & colls [30]. They established that the alterations in expression are significative when the RQ value is over 2, or under 0.5, corresponding to FC +/- 2 values respectively. In the figures, the expression changes are indicated by a diamond. In order to test the significance level of the expression changes, T- student test was performed.

P-Values <0.05 were considered to be statistically significant. The software used to carry out the statistical analysis was SPSS 24.0 (IBM Software).

## Results

### Generation of six new human cell lines of GBM

Fifty-two brain aspirates were collected during this study. All of them were diagnosed as GBM by the Anatomy Pathology Unit. The brain aspirates were processed, and primary cultures were stablished from them. RNA, DNA and cryopreserved cells were obtained systematically from different passages. Finally, six of these primary cultures which had a quickly and stable growth rate and kept their molecular features, were selected for this study. The behaviour of these six primary cultures was similar to those of a cell line. These new cell lines were named HGUE (“Hospital General Universitario de Elche”)-GB (Glioblastoma)-number of culture, for example: HGUE-GB-16. However, for this article they have been named GB+number of culture; for example, GB16, to facilitate easy understanding of Figures and Figures legends.

**Fig 1:**
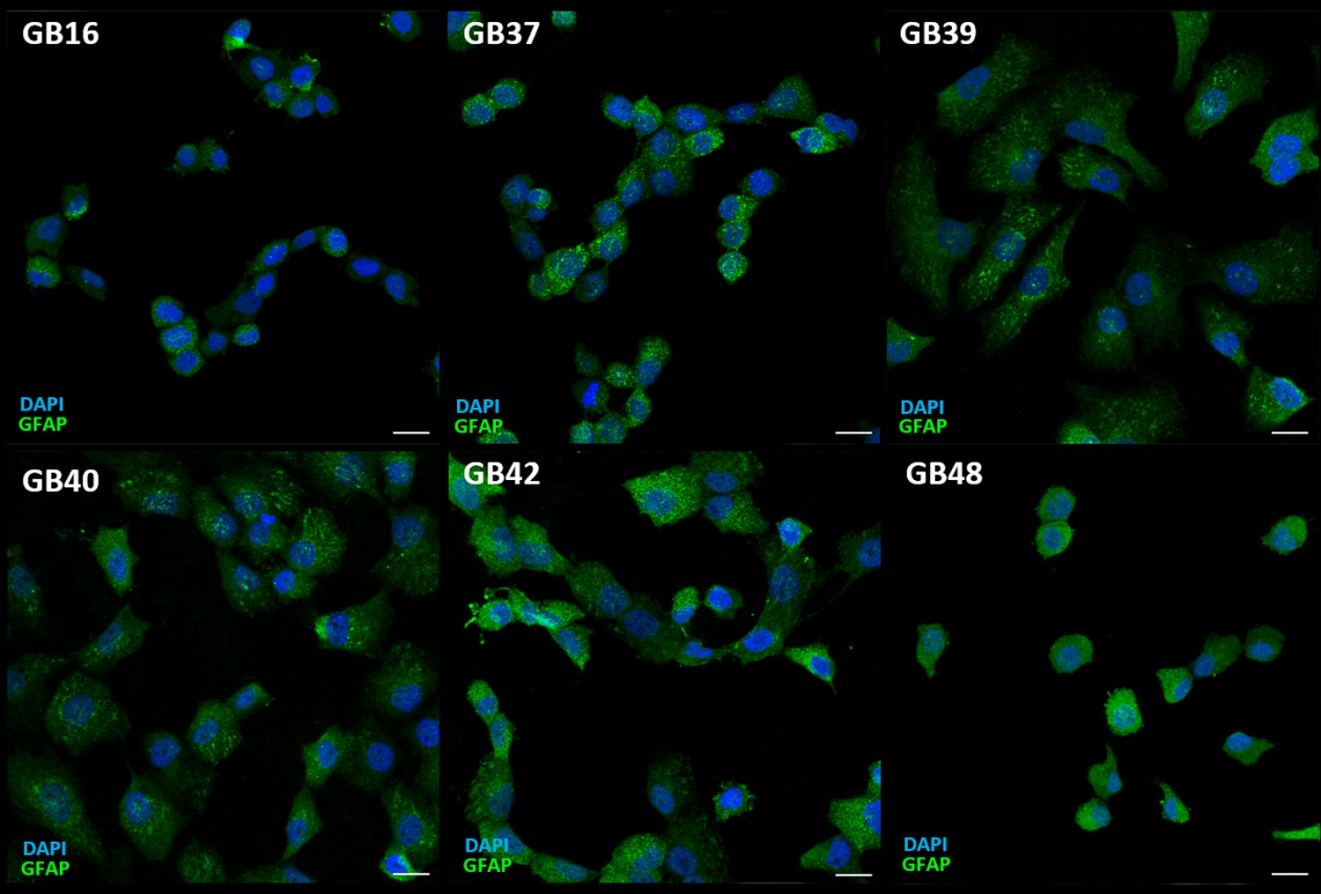
Immunodetection of astrocyte marker (GFAP) in GBM cell lines. Images corresponding to immunolabeling of GFAP (green) in GBM cell lines. Nuclei stained with DAPI are shown in blue. Each bar equals 20 μm.

To confirm whether these six new cell lines had preserved their glial origin, immunocytochemistry assays were performed to detect the expression of glial fibrillar acidic protein (GFAP), since this protein is considered a glial marker, and particularly of astrocytes [31]. Immunolabeling analysis of these cell lines was carried out using a specific antibody anti-GFAP, and subsequently, stained cells were visualized by confocal microscopy. All cell lines studied showed a positive immunoreactivity against GFAP antigen (**Fig 1**), and it was confirmed that all established cell lines had preserved their glial origin.

### The GMB cell lines established belonged to *IDH*-wildtype GBM

**Fig 2:**
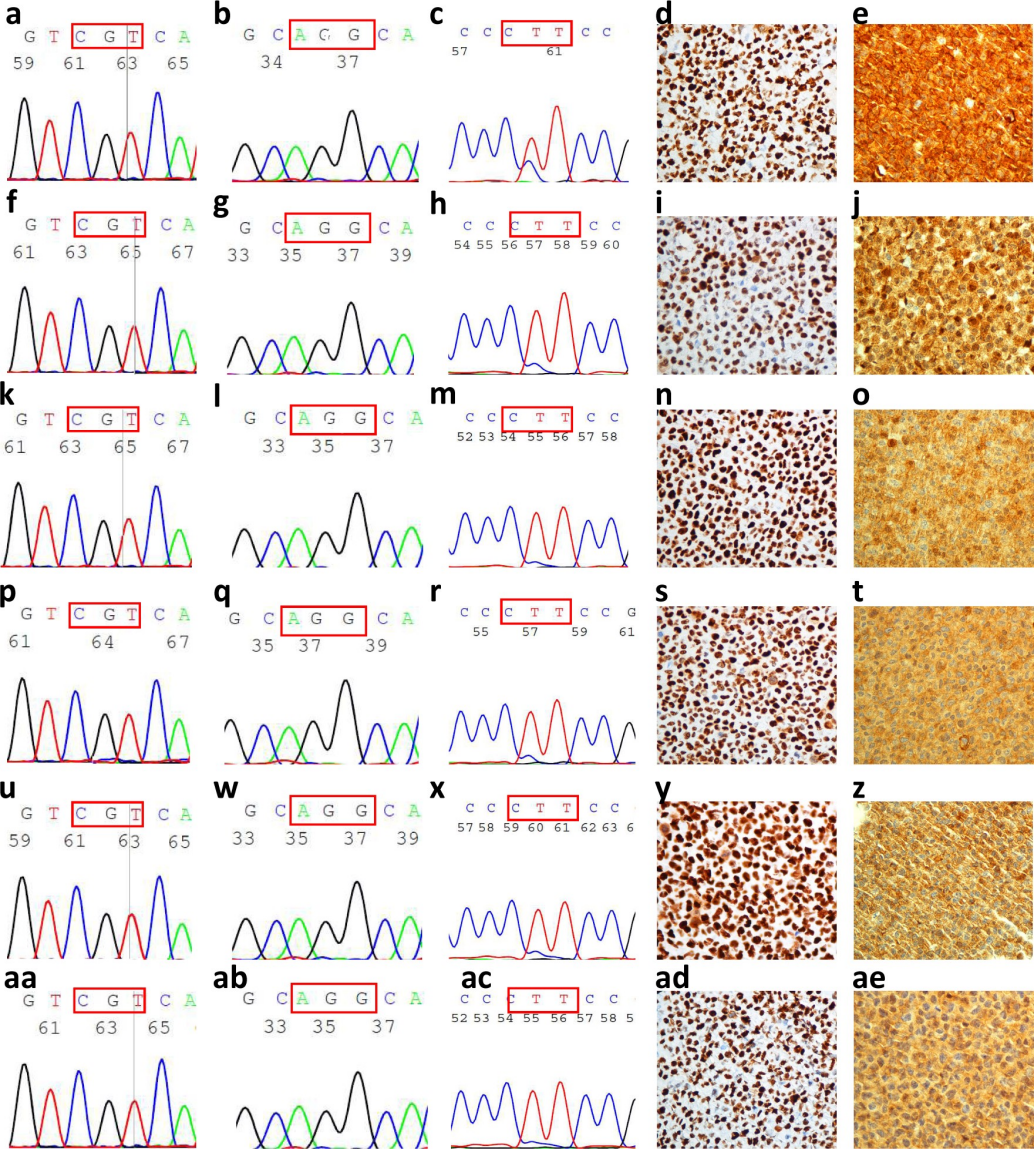
Molecular features of stablished GBM cell lines. Electropherograms of *IDH1* **(a**, **f**, **k**, **p, u, aa**), *IDH2* (**b**, **g**, **l**, **q**, **w**, **ab**), and *TERT* (**c**, **h**, **m**, **r**, **x**, **ac**), and immunocytochemistry images of P53 (**d**, **i**, **n**, **s**, **y**, **ad**) and PTEN (**e**, **j**, **o**, **t**, **z**, **ae**) of all cell lines are shown. The first line corresponds to GB16, the second line to GB37, the third line to GB39, the fourth line to GB40, the fifth line to GB42 and the sixth line to GB48. Red rectangles show the codon which harbours the mutation. The images were made to 40ꓫ.

These new GBM cell lines were classified according to the guidelines proposed by WHO in their 2016 CNS tumours classification [5], the “Hotspot” mutation region sequences of *IDH1*, *IDH2* and *TERT* were determined by Sanger sequencing, and the electropherograms obtained were studied to detect the described mutations: in *IDH1* R132, in *IDH2* R172, and the C225T or C250T *TERT* promoter region [28,32]. No mutations of *IDH1* or *IDH2* were found in the six GBM cell lines (**Fig 2A**, **2F**, **2K**, **2P**, **2U**, **2AA, 2B**, **2G**, **2L**, **2Q**, **2W** and **2AB**, respectively). However, all of them harbour a C250T mutation in the *TERT* promoter (**Fig 2C**, **2H**, **2M**, **2R**, **2X and 2AC**). The GB16 cell line showed this mutation in heterozygosis.

P53 modifications were also analysed by immunocytochemistry, and all established GBM cell lines had alterations in this protein, showed by P53 staining in their nuclei (**Fig 2D**, **2I**, **2N**, **2S**, **2Y and 2AD**). Nevertheless, the cell lines showed positive immunoreactivity to PTEN antigen (**Fig 2E**, **2J**, **2O**, **2T**, **2Z and 2AE**), indicating that *PTEN* expression was conserved.

These results confirm that the established human cell lines are *IDH*-wildtype GBM.

### Methylation status of *MGMT* promoter, expression of *MGMT* mRNA and chemotherapy response

The methylation status of the *MGMT* promoter and its expression have been established as a prognostic and response marker in GBM [33], so both features were determined in these GBM cell lines. Methylation status was studied by MS-MLPA, and the results showed that all the cell lines had methylated the *MGMT* promoter, except the GB16 cell line, which had unmethylated all its CpG islands (**S1 Fig**). Furthermore, the expression of *MGMT* was studied by Q-PCR to associate the methylation status with its expression level. As expected, only the GB16 cell line showed expression of *MGMT* mRNA by Q-PCR, taking into consideration the *MGMT* promoter methylation status. GB16 also exhibited overexpression of *MGMT* mRNA, compared to RNA pool from non-tumoral brain tissue (Fold Change (FC) = 1.7±0.15).

Finally, we characterized the response of these GBM cell lines to BCNU, a drug used in chemotherapy. The most chemosensitive cell line was GB42, as BCNU induced a 49.9% ± 3.7 cell death, followed by the cell lines GB39, GB40 and GB48, which were slightly affected, their viability being reduced by 25.2% ± 1.8, 19.5% ± 1.1 and 27.9% ± 2.1, respectively. The higher rate of survival was achieved by GB16, as only the 12.6% ± 2.3 of its cells were affected by BCNU, so it was considered a GBM resistant cell line to chemotherapy (**S2 Fig**).

### Response to radiotherapy in primary GBM cell lines

**Fig 3:**
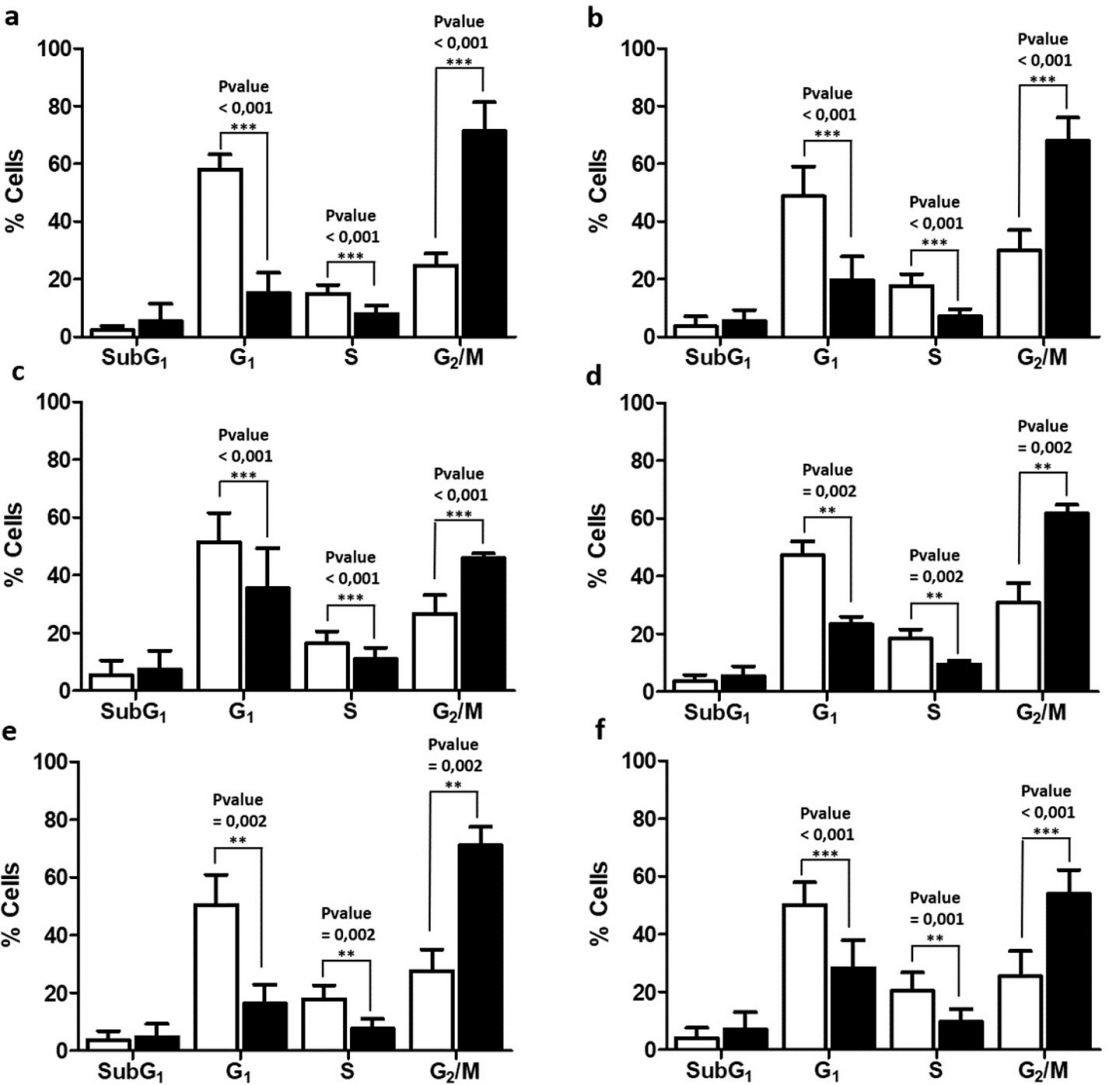
**Cell cycle distribution of GB16 (a), GB 37 (b), GB39 (c), GB40 (d), GB 42 (e) and GB48 (f).** The percentage of cells in each phase of the cell cycle is represented as the average of several experiments (mean ± SD), with the P-value obtained from the non-parametric Mann the Whitney Test (n≥10) superimposed (** P-value< 0.01, *** P-value< 0.001). White bars represented control cells, and black bars irradiated cells.

To evaluate the radiotherapy effect, the cell lines were irradiated to a 7Gy dose (6Gy/min rate). Then, 24 hours after treatment, the cell cycle distribution of different cells (control and irradiated) were compared to determine the response to radiotherapy. Significative changes in the G_1_, S and G_2_/M phases were detected on each cell line due to irradiation. G_1_ and S phases were reduced, and G_2_/M was increased, while SubG_1_ did not show any significant changes (**Fig 3**), suggesting that the first effect of radiotherapy is to arrest cell cycle in the G_2_/M phase of the cell cycle, in GBM cell lines. To determine the fate of cells blocked in phase G_2_/M, viability studies were performed 72h after irradiation. The results showed an increase in the percentage of dead cells similar to the percentage of cells blocked in phase G_2_/M, 24h after irradiation, suggesting that the initially blocked cells end up dying later on (**S3 Fig**).

From the data showed above, the effect of radiotherapy was calculated as the increase of arrested cells in the G_2_/M phase, due to irradiation, plus the increase of cells in SubG_1_, since this phase is associated to cell death (Δ (SubG_1_ + G_2_/M) _IR-CTR_). Any cell line reached a radiotherapy response higher than 50%, the most affected being the GB42 cell line (49.8% ± 1.2 affected cells), and the most radioresistant being the GB39 cell line (21.2% ± 1.6 affected cells). The rest of cell lines showed middle percentages of response.

The radiosensitivity of cell lines was also tested after grew up the cells in serum-free media, and the value of (Δ (SubG_1_ + G_2_/M) _IR-CTR_) for each cell line was similar to the percentages indicated above for the same cell line growing with 10% fetal bovine serum (**S4 Fig**).

### *SOCS1* and *SOCS3* expression in primary GBM

To study the expression profile of *SOCS1* and *SOCS3* in GBM cell lines, RNA from the cell lines in basal state (confluence level 75%, adequate quantity of nutrients and controlled atmosphere) was isolated. Then, a retrotranscription and Q-PCR assay were carried out. FC values were calculated to evaluate the expression differences between GBM and non-tumoral brain tissue.

The GBM cell lines overexpressed *SOCS1* and *SOCS3*, when compared to an RNA pool from non-tumoral brain tissue. The *SOCS1* expression was between 2 and 5 times higher, and the *SOCS3* expression increased in GBM cell lines between 3 and 4.5 times (**S5A Fig**).

Furthermore, a Western blott analysis was carried out to confirm the overexpression of SOCS3 in the GBM cell lines. In this case, total protein extracts from the cell lines were obtained, and then a Western blotting was performed using antibodies to SOCS3 and GAPDH (as endogenous control). All cell lines overexpressed SOCS3 when compared to a protein pool from non-tumoral brain tissue (**S5B Fig**).

### Immunocytochemistry studies of *SOCS1* and *SOCS3* proteins

**Fig 4:**
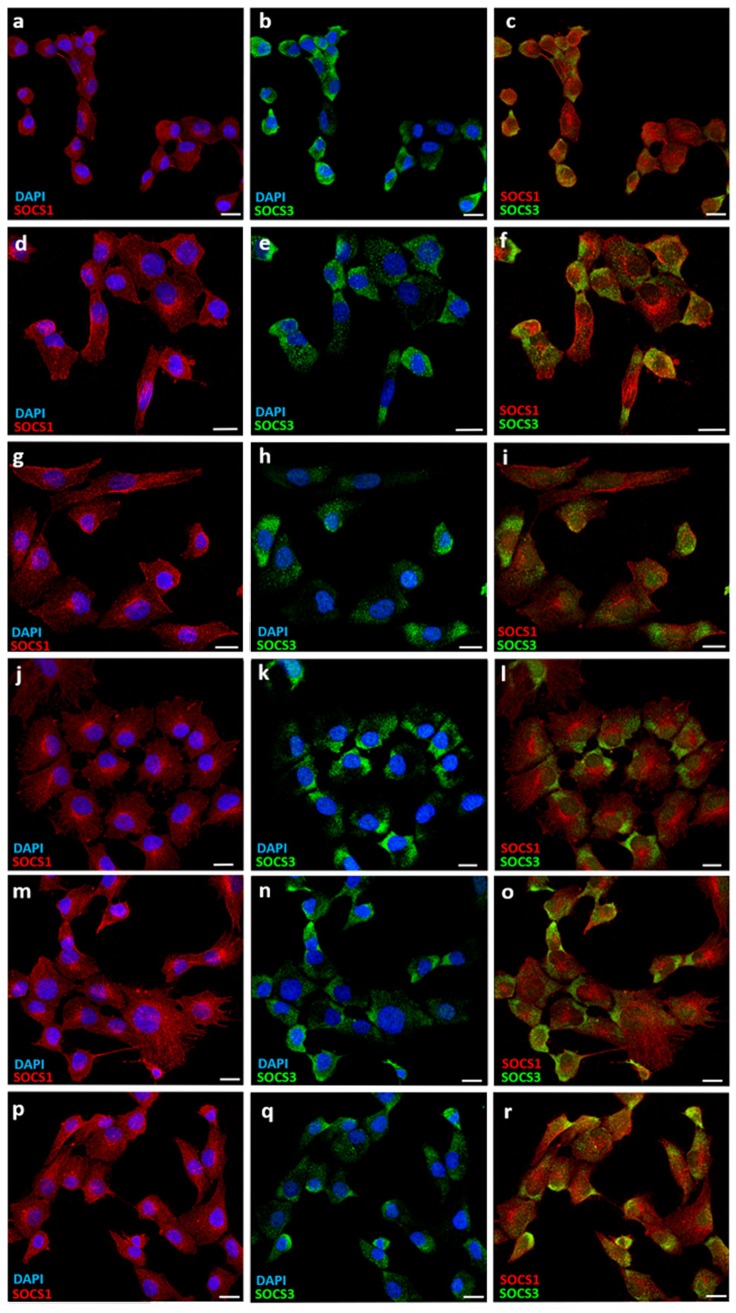
Immunolocalization of SOCS1 and SOCS1 in GBM cell lines. Images show immunolabeling of SOCS1 (red) and SOCS3 (green) of GBM cell lines: GB16 **(a, b, c)**, GB37 **(d, e, f)**, GB39 **(g, h, i)**, GB40 **(j, k, l)**, GB42 **(m, n, o)**, GB48 **(p, q, r)**. Nuclei stained with DAPI are shown in blue. Each bar equals 20 μm.

To confirm the expression data of *SOCS1* and *SOCS3* obtained by Q-PCR, immunocytochemistry assays to detect the presence of SOCS1 and SOCS3 proteins were performed in GBM cell lines. Double immunolabeling was carried out using anti-SOCS1 and anti-SOCS3 antibodies. The six GBM cell lines showed positive immunostaining for both proteins, which indicates that mRNA expression was correlated with protein presence. SOCS1 and SOCS3 proteins appeared in the cytoplasm, without apparently association to any organelle and no presence in the nuclei. The double immunolabeling did not show colocalization of these proteins in the cytoplasm (**Fig 4**).

### Effect of the radiotherapy exposure on the *SOCS1* and *SOCS3* expression at mRNA level

**Fig 5:**
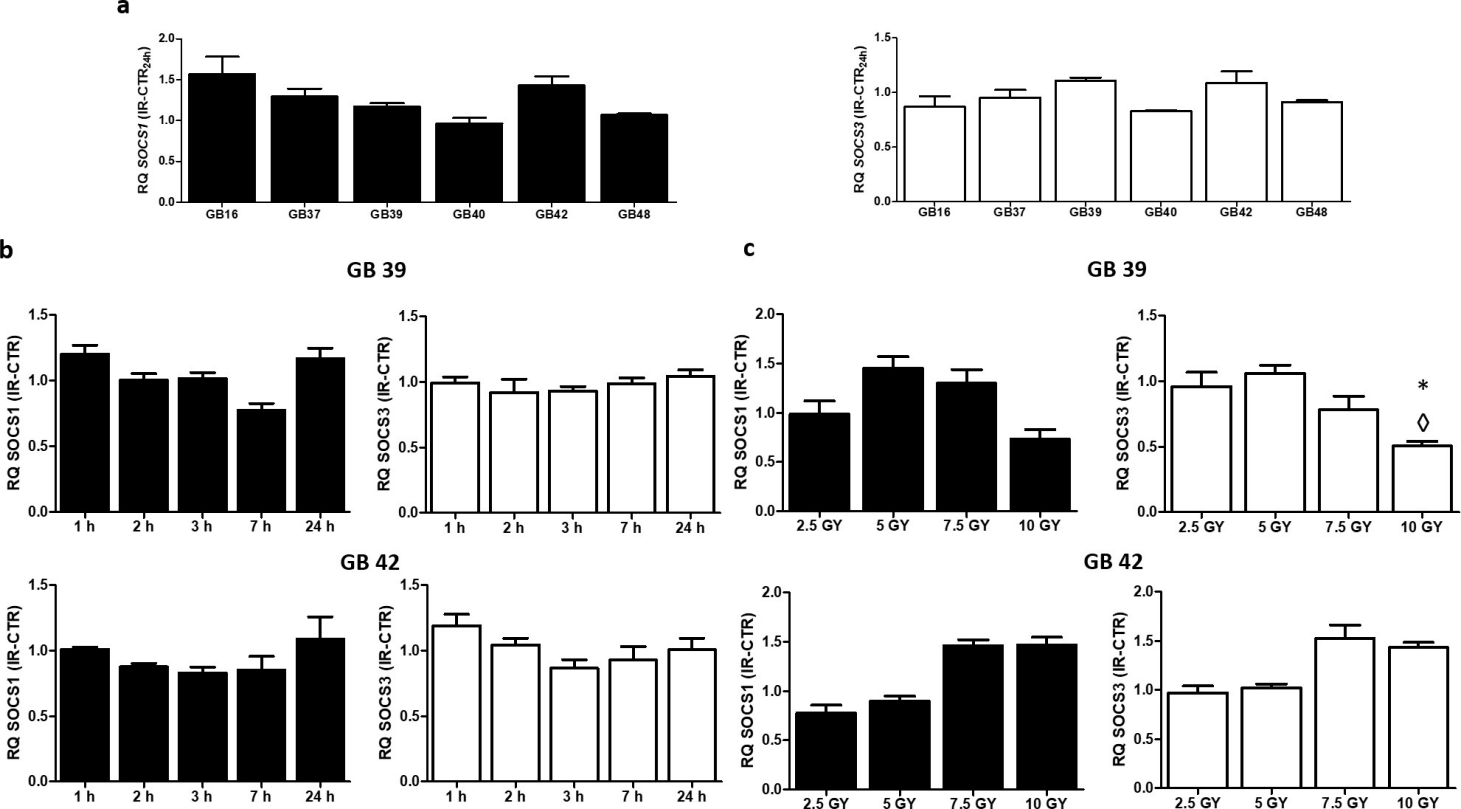
Expression of *SOCS1* and *SOCS3* after radiotherapy in GBM cell lines. Values of RQ ± SD 24 hours post-irradiation of *SOCS1* and *SOCS3* in all cell lines (**a**), and values of RQ ± SD obtained by time course (**b**) and dose-response (**c**) studies in GB 39 and in GB42. Black bars correspond to *SOCS1* RQ values, and white bars to *SOCS3* RQ values. ◊ means changes in expression. * Indicate a P-value< 0.05 (n≥3).

In order to study the effect of radiotherapy on *SOCS1* and *SOCS3* mRNA expression, GBM cell lines were exposed to 7Gy, and 24 hours after irradiation cells were harvested, and total RNA was isolated. In parallel, control non-irradiated cells were collected to be used as an expression control. No significant changes were observed in *SOCS1* and *SOCS3* mRNA levels after irradiation (**Fig 5A**).

To determine whether *SOCS1* and *SOCS3* mRNA expression was significative affected after exposure to radiotherapy, time-course and dose-response experiments were performed. In this case, the study was carried out in the most radioresistant cell line (GB39), and in the most radiosensitive one (GB42). Non-treated cell lines were used as expression control, and RQ values were calculated from them. Changes in *SOCS1* and *SOCS3* mRNA expression were not detected in GB39 nor GB42 at different times (**Fig 5B**). Moreover, changes on *SOCS1* and *SOCS3* expression were undetected in dose-response experiments (**Fig 5C**), with the only exception of the GB39 cell line, where its expression was reduced by half, in comparison with the control (RQ = 0.5 ±0.03) at the highest radiotherapy dose.

### Reduction of *SOCS1* and *SOCS3* mRNA expression by small interfering RNA (siRNA) and its effect on radiotherapy resistance

To determine whether *SOCS1* and/or *SOCS3* play a role in GBM radioresistance, *SOCS1* and *SOCS3* expression was inhibited using siRNA. First, we studied that transfection with non-specific siRNA (siRNA.NS) did not altered *SOCS1* and *SOC3* expression, nor cell cycle distribution. The results did not show significative differences between control and siRNA.NS transfected cells in any cases (**S6 Fig**).

**Fig 6:**
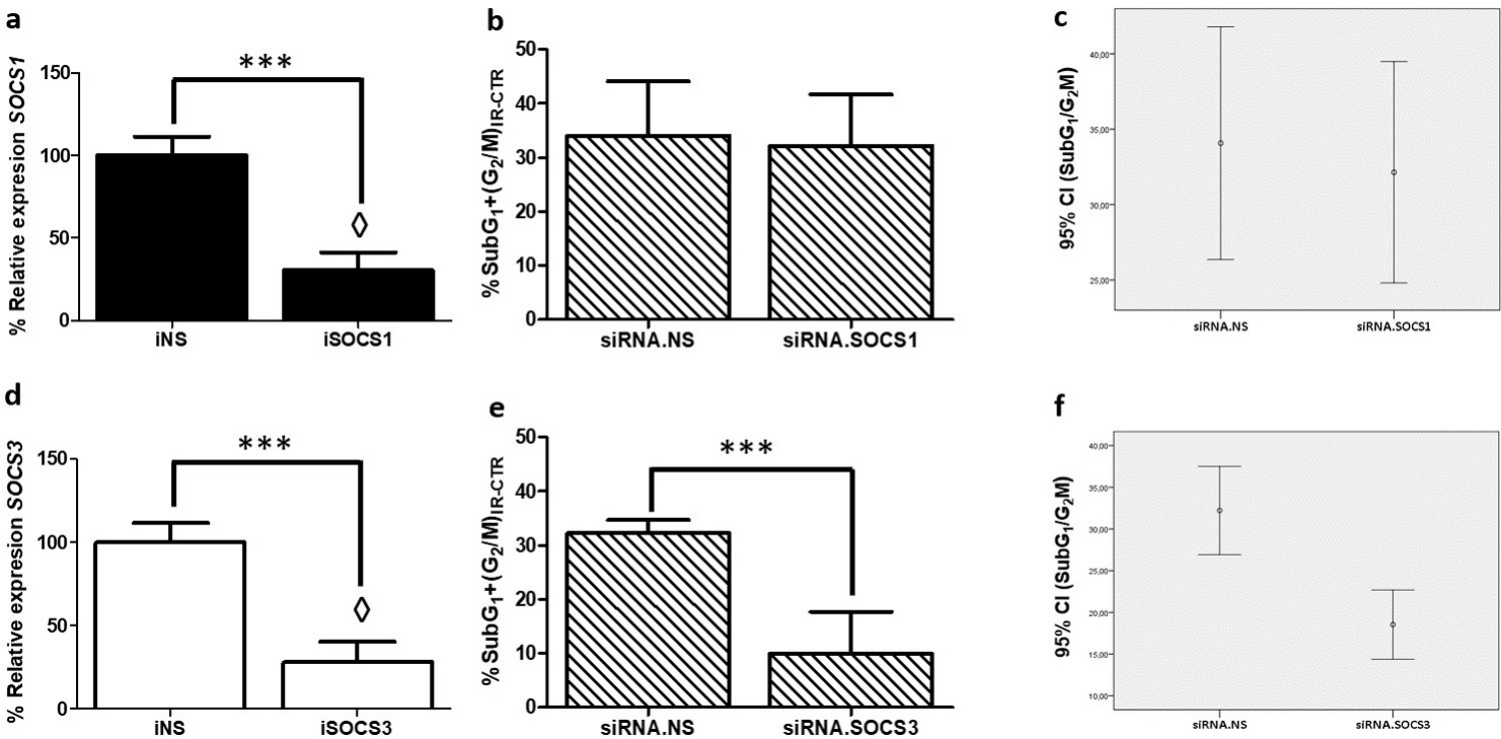
*SOCS1* and *SOCS3* interfering assay and its biological effect on radiotherapy response. Mean ± SD of relative mRNA expression of *SOCS1* **(a)** and *SOCS3* **(d)** in siRNA.NS, siRNA.SOCS1 and siRNA.SOCS3 transfected cells. Mean Percentage of arrested cells in SubG_1_ and G_2_/M phases due to radiotherapy in siRNA.NS and siRNA.SOCS1 **(b)**, and in siRNA.NS and siRNA.SOCS3 **(e)**. Representation of 95% confidence interval of radiotherapy response in siRNA.NS and siRNA.SOCS1 **(c)**, and in siRNA.NS and siRNA.SOCS3 transfected cells **(f)**. ◊ means changes in expression. *** Indicate P-value<0.001 (n≥3).

Subsequently, parallel assays of expression pre-irradiation and cell cycle post-irradiation were carried out. In this case, we compared the mRNA expression between siRNA.NS transfected cells and siRNA.SOCS1 or siRNA.SOCS3 transfected cells, which demonstrated that expression of *SOCS1* and *SOCS3* was significative reduced. Cell cycle distribution was also studied in each sample (siRNA.NS, siSOCS1, siSOCS3) in order to evaluate the effect of radiotherapy (**Fig 6**).

The mRNA expression of *SOCS1* was reduced by 70% in siRNA.SOCS1 transfected cells when compared to siRNA.NS (**Fig 6A**). However, the effect of irradiation was the same on siRNA.NS and siRNA.SOCS1 cells (**Fig 6B and 6C**). Therefore, the radiotherapy response was not affected by *SOCS1* expression reduction.

The expression of *SOCS3* was reduced by 73% in siRNA.SOCS3 transfected cells (**Fig 6D**). Furthermore, the irradiation caused a significative differential effect in siRNA.NS and siRNA.SOCS3 transfected cells (Pvalue < 0.001). The siRNA.SOCS3 transfected cells were more radioresistant than the cells transfected with siRNA.NS, since their percentage of cells in the SubG_1_ and G_2_/M phases post-irradiation was smaller than the number of cells in these phases in the siRNA.NS transfected cells (**Fig 6E and 6F**). These data reveal that *SOCS3* expression inhibition provides resistance to radiotherapy in GBM cell lines.

### *SOCS1* and *SOCS3* mRNA expression in clonal radioresistant populations

Clonal radioresistant populations were obtained from the most radioresistant cell line (GB39), and from the most radiosensitive cell line (GB42). After an accumulated 45Gy (5Gy/week) dose treatment, 10 clonal survival populations were isolated from GB39, and 6 from GB42. Finally, 6 clonal survival populations from GB39, and 1 from GB42 were obtained.

**Fig 7:**
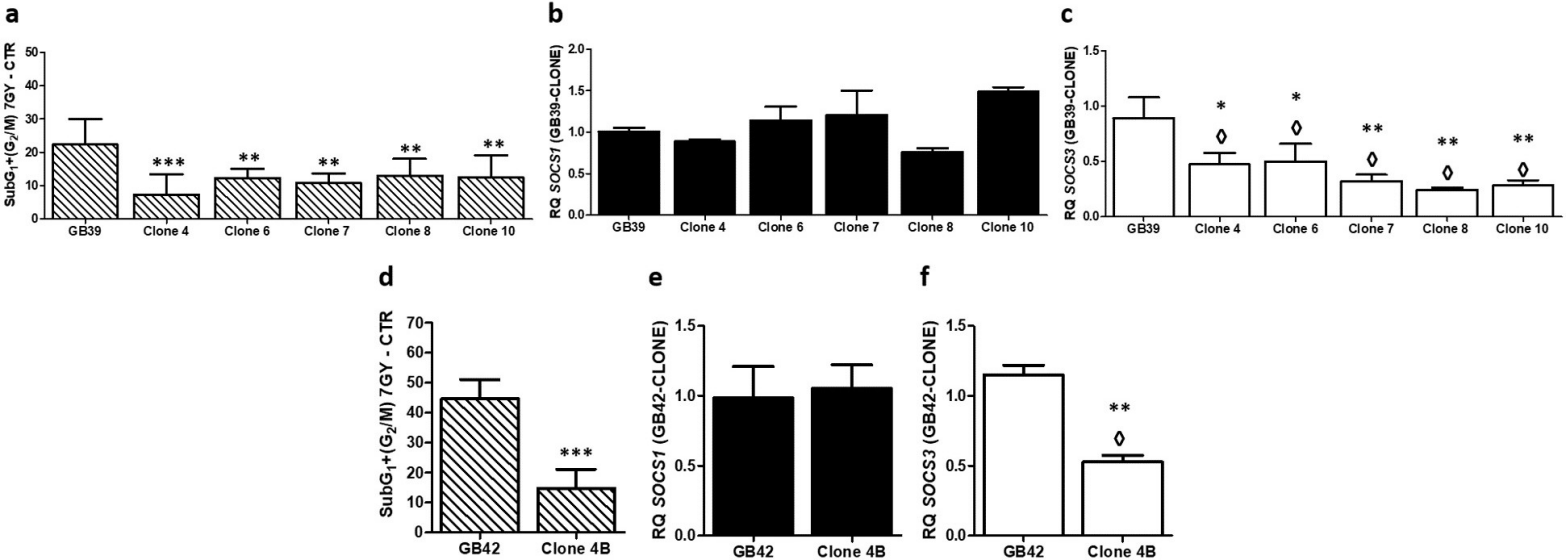
Cell cycle analysis, and *SOCS1* and *SOCS3* expression on the clonal isolated resistant populations and parental cell lines. Data are presented as the mean percentage ± SD of arrested cells in SubG_1_ and G_2_/M phase due to radiotherapy. arrested **(a,d)**. Mean ± SD of *SOCS1* mRNA expression **(b, e)** and *SOCS3* **(c, f)** in parental cell lines and clonal populations. ◊ means changes in expression. * Indicate P-value<0.05, ** Indicate P-value<0.01, and *** indicate P-value<0.001 (n≥3).

As expected, cell cycle analysis showed that the isolated clonal populations were more radioresistant than their parental cell line (**Fig 7A and 7D**). The expression of *SOCS1* and *SOCS3* at mRNA level was determined in all of them. While the expression of *SOCS1* was unmodified (**Fig 7B and 7E**), the expression of *SOCS3* was significantly lower in all clonal radioresistant populations in contrast to their parental cell lines (**Fig 7C and 7D**). These results reinforce the data obtained by siRNA.

### Induction of *SOCS3* expression and its role in the radiotherapy response

#### Induction of *SOCS3* expression by a heterologous promoter

The study of radiotherapy sensitization was performed in the most radioresistant GBM cell line (GB39). In order to achieve the upregulating of *SOCS3*, this cell line was transfected with 15 ng or 25 ng of synthetic plasmid, which contains the *SOCS3* ORF under a heterologous promoter control.

**Fig 8:**
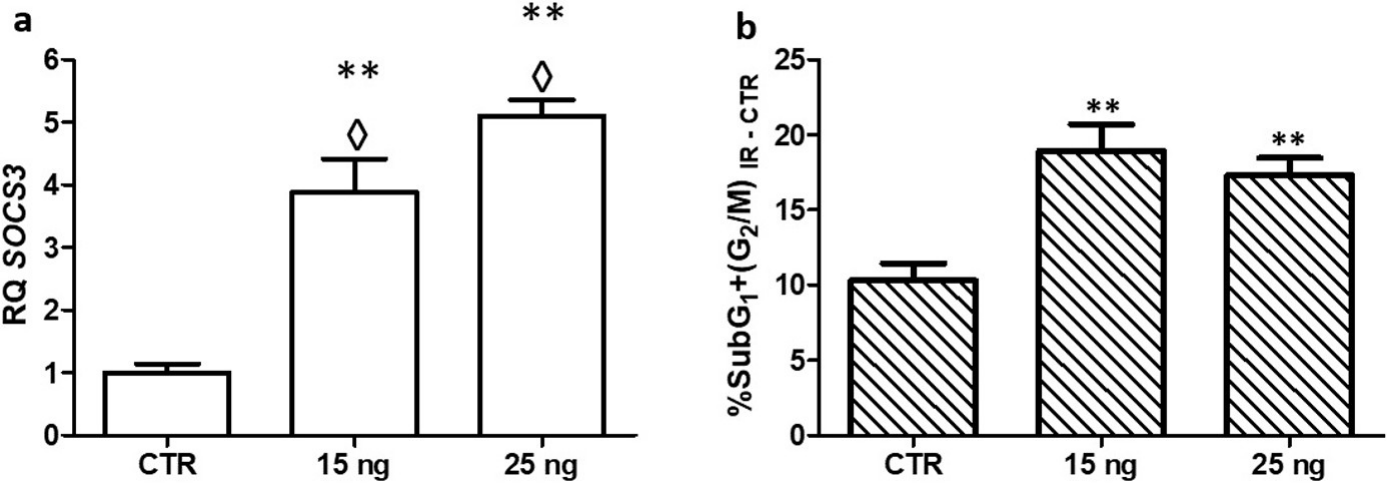
Induction of *SOCS3* expression by a heterologous promoter. Mean ± SD values of relative expression of *SOCS3* **(a)** and cellular mean percentages of cells in SubG_1_ and G_2_/M phases **(b)**. CTR: sample transfected with control plasmid, 15ng and 25ng: cells transfected with 15 and 25 ng of *SOCS3* synthetic plasmid, respectively. ◊ means changes in expression. ** Indicate P-value<0.01 (n≥3).

The increase of *SOCS3* mRNA expression was evaluated by Q-PCR, and the data obtained confirmed that cells transfected with 15ng and 25 ng of *SOCS3* plasmid increased the expression of *SOCS3*, when compared to cells transfected with control plasmid. The cells transfected with 25 ng of plasmid showed four times increase in *SOCS3* mRNA level, and the cells transfected with 15 ng showed three times increase (**Fig 8A**), which suggest that the amount of plasmid correlates with the increase of gene expression.

In relation to the radiotherapy effect, cells with higher *SOCS3* mRNA expression showed a greater response to irradiation, compared to control cells. The percentage of cells in SubG_1_ and G_2_/M phases was twice as much in cells transfected with *SOCS3* plasmid than in cells transfected with control plasmid (**Fig 8B).** Therefore, when *SOCS3* is upregulating the culture showed a better response to radiotherapy.

#### Induction of endogenous *SOCS3* expression by TSA treatment

**Fig 9:**
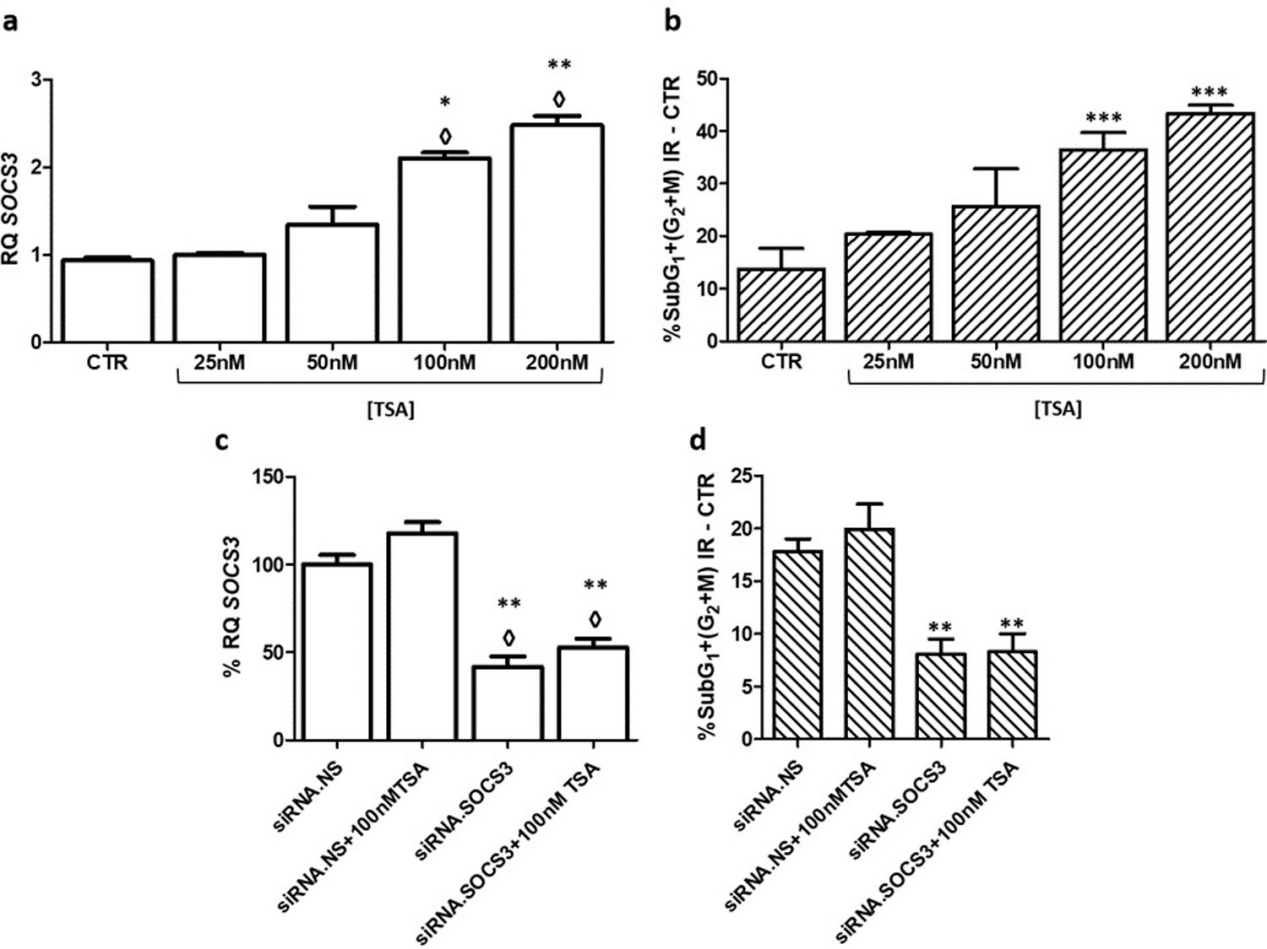
TSA pre-treatment and its effect upon cell cycle and *SOCS3* expression. White bars show mean ± SD of *SOCS3* mRNA expression in the different experiments (**a**,**c**). Striped bars represent mean percentages ± SD of cells in SubG_1_ and G_2_/M phases (**b**,**d**). ◊ means changes in expression ** Indicate P-value<0.01, *** Indicate P-value<0.001 (n≥3).

Finally, we wondered whether increasing endogenous *SOCS3* expression by an alternative treatment, such as the iHDAC named TSA, will be able to sensitize the most radioresistant established cell line to radiotherapy. To test this hypothesis, the cells were pre-treated for 16 hours with different doses of TSA, in a range from 25–200 nM. Then, the expression of *SOCS3* mRNA was determined just before the irradiation, and the effect of radiotherapy was determined 24 hours after the treatment by cell cycle analysis. The *SOCS3* mRNA expression was increased by TSA treatment in a dose-depended manner (**Fig 9A**), and the radiosensitive of GB39 cell line also increased in the same way (**Fig 9B**).

To demonstrate that the TSA-induced increase in radiosensitivity showed in the radioresistant cell line was due to the increase in *SOCS3* expression, a siRNA experiment blocking *SOCS3* expression was performed, with or without TSA pre-treatment, keeping on this way the *SOCS3* mRNA levels downregulated. In these conditions, the radiosensitization effects of TSA disappeared (**Fig 9C and 9D**).

The effect of TSA was also tested in the most radiosensitivity cell line (GB42) following the same conditions used for GB39. In this case, the endogenous levels of *SOCS3* and the percentages of SubG_1_+G_2_/M remained unchanged for all TSA concentrations tested (**S7 Fig**).

## Discussion

The progression of GBM is fast and difficult to control, due to its chemo and radioresistance. Hence, it is very important to obtain accurate models to study this type of cancer. For that purpose, there are many cell lines which mimic GBM, such as U87MG or LN229 (ATCC collection), but these cell lines are far away from the patient state and they usually have been modified to achieve an immortalized status. The six new human GBM cell lines established in this study resemble more the patient state, because not only they were originated from them but also, they preserve their glial nature and molecular features of the initial tumour and they lack any artificially induced modification. Other advantage of using these cell lines from primary cultures is the possibility to recreate their progression and go back and forth in their development to study gene expression or physiological properties, as DNA, RNA and cryopreserved cells have been stored at different passages.

The molecular studies of these cell lines have confirmed that they were included in the *IDH*-wildtype GBM, which is the most aggressive GBM group, according to 2016 WHO SNC classification. Moreover, they harbour *TERT* mutations in their promoters, and alterations in P53, two characteristics that support the fact that they are indeed primary GBM [5]. To date, the only *IDH*-wildtype GBM well characterized according to WHO are these cell lines, so they can be used as a proper model of primary GBM to study the malignant features of these tumours.

In relation to chemotherapy, all these cell lines showed a middle response to BCNU treatment, reinforcing their classification as primary GBM. The most resistant cell line affected only by 12.6% ± 2.3, was GB16. Epigenetic results indicated that this cell line has the *MGMT* promoter methylated, and unlike the rest of cell lines, it expresses this gene at the mRNA level. Furthermore, we demonstrated the relationship between *MGMT* promoter methylation status and chemoresistance, which has been postulated in many previous studies [33]. However, the low response obtained by the other cell lines suggest that resistance to alkylating drugs must be regulated by other mechanisms not confirmed as yet, such as the presence of tumour stem cells, capable of resisting treatment and replace dead cells, already described in other types of cancers[34,35].

Moreover, radiotherapy affected the distribution of the cells in the different phases of the cell cycle, reducing the cellular percentage in the G_1_ and S phases, and increasing the percentage of cells in G_2_/M phase. Thus, the statistically significant increase of arrested cells in the G_2_/M phase was stablished as a hallmark to determine the radiotherapy effect on these cell lines. According to this value, the GBM cell lines showed a low radiotherapy response, and never reached more than 50%, indicating again that they belong to the worst prognostic group.

*SOCS* genes have been associated with carcinogenesis and tumour progression [36]. Particularly, *SOCS1* and *SOCS3* have been postulated as candidates to regulate the radio and chemoresistance acquisition in other types of cancer, such as prostate cancer [37,38], or cervix cancer[20], but there is a lack of studies regarding the role of *SOCS1* and *SOCS3* in radiotherapy resistance in GBM. These genes inhibit the cytokine response in many cell types, including CNS, and are involved in the immune response [39]. SOCS proteins also modulate the JAK-STAT pathway, which is involved in many cellular processes, such as cell migration, cell proliferation and apoptosis. SOCS1 and SOCS3 have three different ways to inhibit the JAK/STAT pathway: first, they can bind directly to JAKs proteins, silencing their activity; second, SOCS can bind and block STAT proteins; and finally, they can establish connections with the BC complex and Cullin 5, promoting JAK ubiquitination [14].

*SOCS1* and *SOCS3* were overexpressed in the six GBM cell lines analysed. This increase in mRNA expression has been previously reported in breast cancer where SOCS family plays a role in tumour proliferation [40]. The expression of these genes remained constant after different doses and radiotherapy periods, yet there are no studies in GBM and other cancers related to expression changes of these genes due to radiotherapy. However, a similar study in sarcoma has identified several genes implicated in chemoresistance [41]. Therefore, it is relevant to perform these studies to dilucidate the expression changes involved in radioresistance. On the other hand, the inhibition of *SOCS1* expression by a siRNA did no change the radiotherapy response in any of the six cell lines, and the isolated clonal resistant populations did not show any modification in the level of *SOCS1* mRNA expression, indicating that *SOCS1* is not directly related to radioresistance acquisition in GBM, as it was postulated by Zhou *et al* [18]. The reason for the discrepancy is unknown, but their studies were carried out only in a GBM cell line (U87) and *SOCS1* was ectopically expressed, because it was silenced in U87 cells by promoter methylation [18]. Meanwhile, our data have been obtained in six different new established cell lines where the *SOCS1* promoter is not methylated. In contrast, the inhibition of *SOCS3* expression by siRNA significantly increased radioresistance of the six cell lines studied, and also there was a downregulation of *SOCS3* mRNA levels in all the isolated clonal radioresistant populations, suggesting again that *SOCS3* plays a key role in GBM radioresistance. Zhou *et al* indicated that the depletion of *SOCS3* induced radiosensitivy, though in their study *SOCS3* wasn’t directly silenced, and radiotherapy response was tested only in U87 cell line which expressed a phosphorylation-deficient DN STAT3 allele [18], and this mutation could affect the whole JAK-STAT pathway. Moreover, our results about *SOCS3* are in agreement with previous studies that have hypothesised the relationship between the methylation of the *SOCS3* promoter and poor prognosis in GBM [17], and its low expression and worse response in other cancers [19,20]. Furthermore, low levels of *SOCS3* could lead to an increase in the JAK/STAT pathway activation, which would contribute to acquisition of features of tumour invasion and metastasis [15].

To further support the implication of *SOCS3* in radioresistance acquisition, *SOCS3* expression was induced under a heterologous promoter in the most radioresistant established cell line (GB39). Upregulation of *SOCS3* produced radiosensitization in this cell line demonstrating again, that *SOCS3* participates in GBM radioresistance. Finally, the endogenous expression of *SOCS3* was also upregulated by TSA treatment, and again the most radioresistant cell line showed an increase in its radiosensitivity, in a TSA dose-depended way. This effect of TSA disappeared when the increase in *SOCS3* expression induced by TSA was blocked by SOCS3.siRNA. Previous studies in myeloproliferative neoplasms and colorectal cancer, indicated that TSA treatment increases the *SOCS3* expression, and this increase repressed the cell growth in both type of cancers [25,42]. These publications also reveal the increase of *SOCS3* expression was associated to a lower activation of JAK/STAT pathway, reinforcing the key role of *SOCS3* and JAK/STAT pathway in radioresistance acquisition proposed in this work.

The data obtained in this study demonstrate the implication of *SOCS3* expression in radiotherapy resistance acquisition in GBM, so the downregulation of *SOCS3* expression by siRNA increased the radiotherapy resistance in all the cell lines tested. In the same sense, a statistically significant downregulation of *SOCS3* mRNA was observed in all the isolated radioresistant clonal population, when compared with the parental cell line. On the other hand, the induction of *SOCS3* expression under a heterologous promoter in the most resistant established cell line induced its radiosensitization. Finally, the pre-treatment with TSA in the stablished cell lines produced an increase in *SOCS3* expression levels and a parallel radiosensitization of these cells. Interestingly, the effects of TSA are completely abrogated when the increase in *SOCS3* mRNA produced by this drug is blocked by SOCS3 siRNA.

Taken together, the results probe that *SOCS3* plays a key role in radioresistance acquisition in GBM, and reveal the possible implication of the JAK/STAT pathway, which is inhibited by SOCS, in response to radiotherapy. These findings show that *SOCS3* and its signal transduction pathway could be considered as a source of putative new targets to overcome GBM radioresistance.

## Supporting information

S1 Fig **Methylation status of *MGMT* promoter of GBM cell lines: GB16 (a), GB37 (b), GB39 (c), GB 40 (d), GB42 (e), GB 48 (f).** Black bars show control methylated CpG islands. Yellow lines show the threshold to consider a methylated CpG island, following the Jeuken et al [29] criteria. Red arrows, on different colours bars, indicate the CpG islands of *MGMT*. In the abscissa axis, all promoter of genes studied by SALSA MS-MLPA ME011.B1 Mismatch Repair Genes kit are shown.
(TIF)

S2 Fig BCNU effect. Bars show the percentage ± SD of cell death in each cell line.
(TIF)

S3 Fig Radiotherapy effect 72 hours post-treatment. Bars show the percentage ± SD of cell death in each cell line.
(TIF)

S4 Fig Radiotherapy effect in cell lines grown in serum-free media. Bars show the effect of radiotherapy as the (Δ (SubG_1_ + G_2_/M) _IR-CTR_) ± SD.
(TIF)

S5 Fig (a) Expression profile of SOCS1 and SOCS3 in primary GBM cell lines. Fold change (FC) values ± SD of SOCS1 mRNA expression (black bars), and SOCS3 mRNA expression (white bars) are shown for each cell line represented in the X-axis. (b): Western blot analysis of SOCS3 expression on total protein extracts from established cell lines. The molecular sizes of the bands are shown to the right.
(TIF)

S6 Fig Analysis of siRNA transfection effect by itself. Representation of the 95% confidence interval of % of cells in the SubG_1_ and G_2_/M phases **(a)** and mRNA expression **(b)** for control (CTR) and transfected with non-specific siRNA (siRNA.NS) cells.
(TIF)

S7 Fig TSA pre-treatment and its effect upon cell cycle and *SOCS3* expression. White bars show mean ± SD of *SOCS3* mRNA expression in the different experiments (**a**). Striped bars represent mean percentages ± SD of cells in SubG_1_ and G_2_/M phases (**b**). (n≥3).
(TIF)
