# Correction: Radiotherapy resistance acquisition in Glioblastoma. Role of *SOCS1* and *SOCS3*

**DOI:** 10.1371/journal.pone.0215714

**Published:** 2019-04-16

**Authors:** Maria Paz Ventero, Maria Fuentes-Baile, Cristina Quereda, Elizabeth Perez-Valeciano, Cristina Alenda, Pilar Garcia-Morales, Danilo Esposito, Pilar Dorado, Victor Manuel Barbera, Miguel Sacedan

There is an error in the XML that is causing the ninth author’s name, Victor Manuel Barbera, to be indexed incorrectly in PubMed. The correct name should be: VM Barbera.
