# Supplementary figures and images for: Radiotherapy resistance acquisition in Glioblastoma. Role of SOCS1 and SOCS3
Source: PLoS One. 2019 Feb 27;14(2):e0212581. doi: 10.1371/journal.pone.0212581 (PMC6392282; doi:10.1371/journal.pone.0212581)

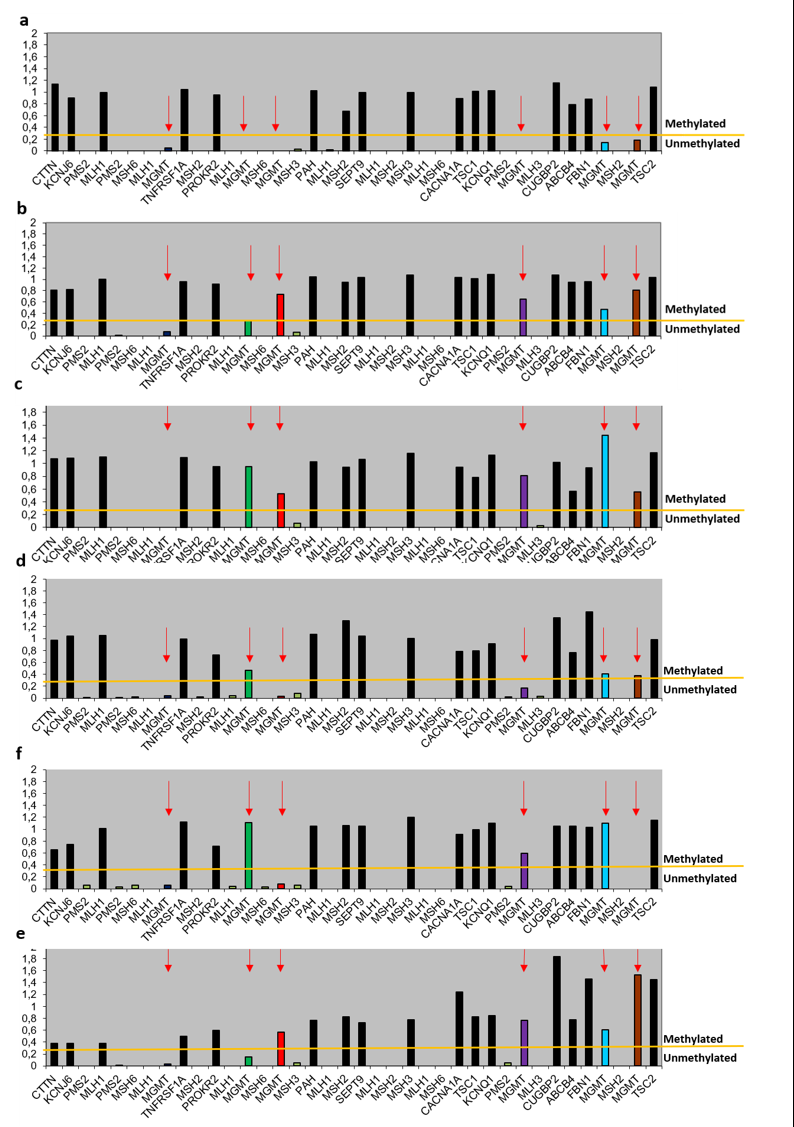

Supplement: S1 Fig — Methylation status of MGMT promoter of GBM cell lines: GB16 (a), GB37 (b), GB39 (c), GB 40 (d), GB42 (e), GB 48 (f). Black bars show control methylated CpG islands. Yellow lines show the threshold to consider a methylated CpG island, following the Jeuken et al [29] criteria. Red arrows, on different colours bars, indicate the CpG islands of MGMT. In the abscissa axis, all promoter of genes studied by SALSA MS-MLPA ME011.B1 Mismatch Repair Genes kit are shown. (TIF) [file pone.0212581.s001.tif]

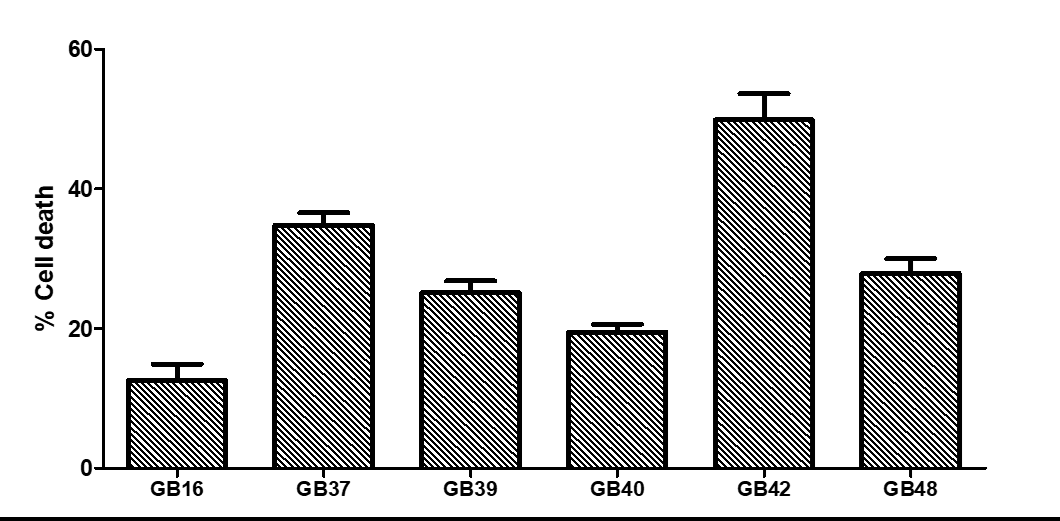

Supplement: S2 Fig — Bars show the percentage ± SD of cell death in each cell line. (TIF) [file pone.0212581.s002.tif]

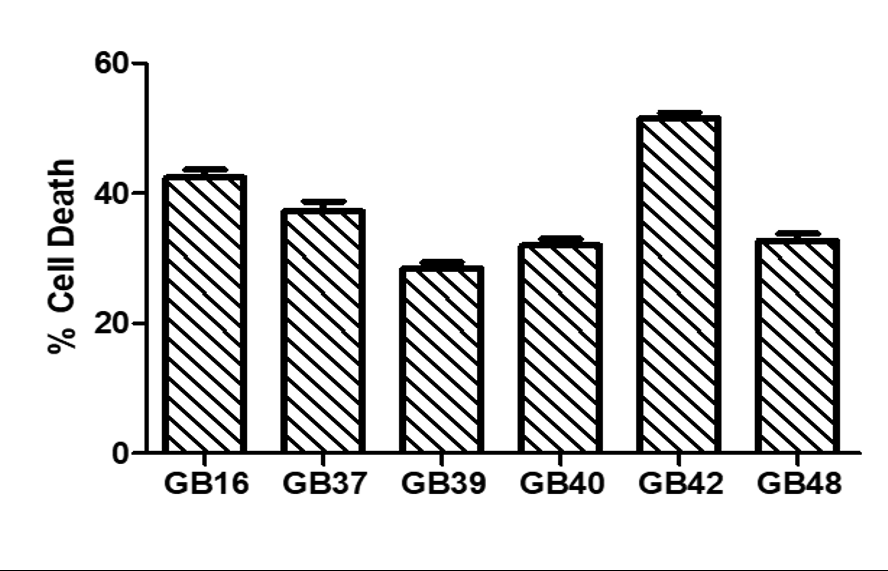

Supplement: S3 Fig — Bars show the percentage ± SD of cell death in each cell line. (TIF) [file pone.0212581.s003.tif]

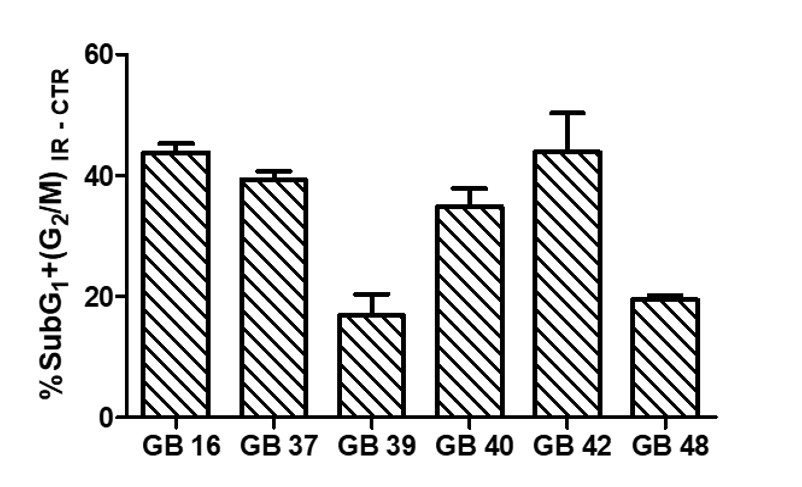

Supplement: S4 Fig — Bars show the effect of radiotherapy as the (Δ (SubG1 + G2/M) IR-CTR) ± SD. (TIF) [file pone.0212581.s004.tif]

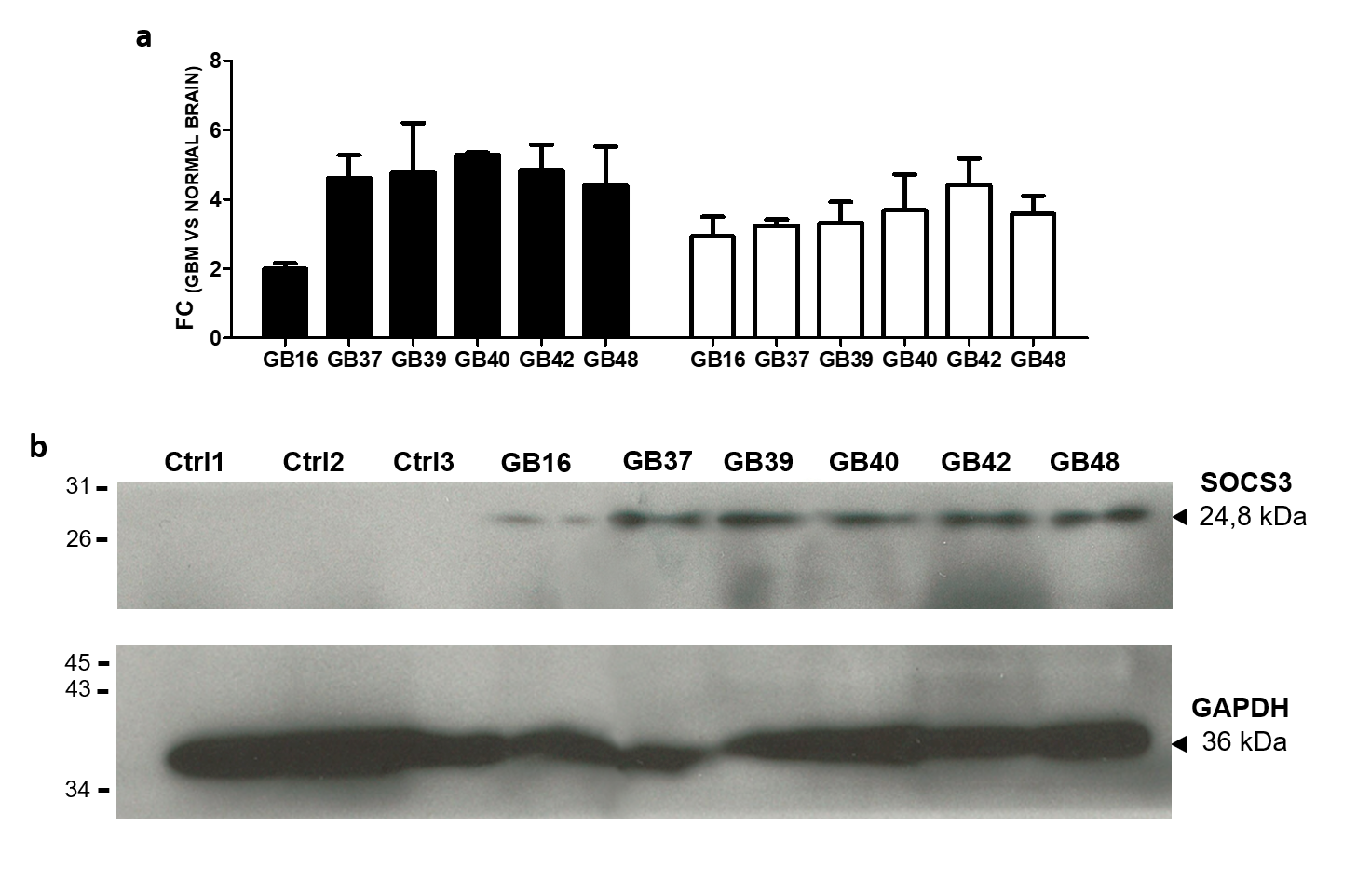

Supplement: S5 Fig — (a) Expression profile of SOCS1 and SOCS3 in primary GBM cell lines. Fold change (FC) values ± SD of SOCS1 mRNA expression (black bars), and SOCS3 mRNA expression (white bars) are shown for each cell line represented in the X-axis. (b): Western blot analysis of SOCS3 expression on total protein extracts from established cell lines. The molecular sizes of the bands are shown to the right. (TIF) [file pone.0212581.s005.tif]

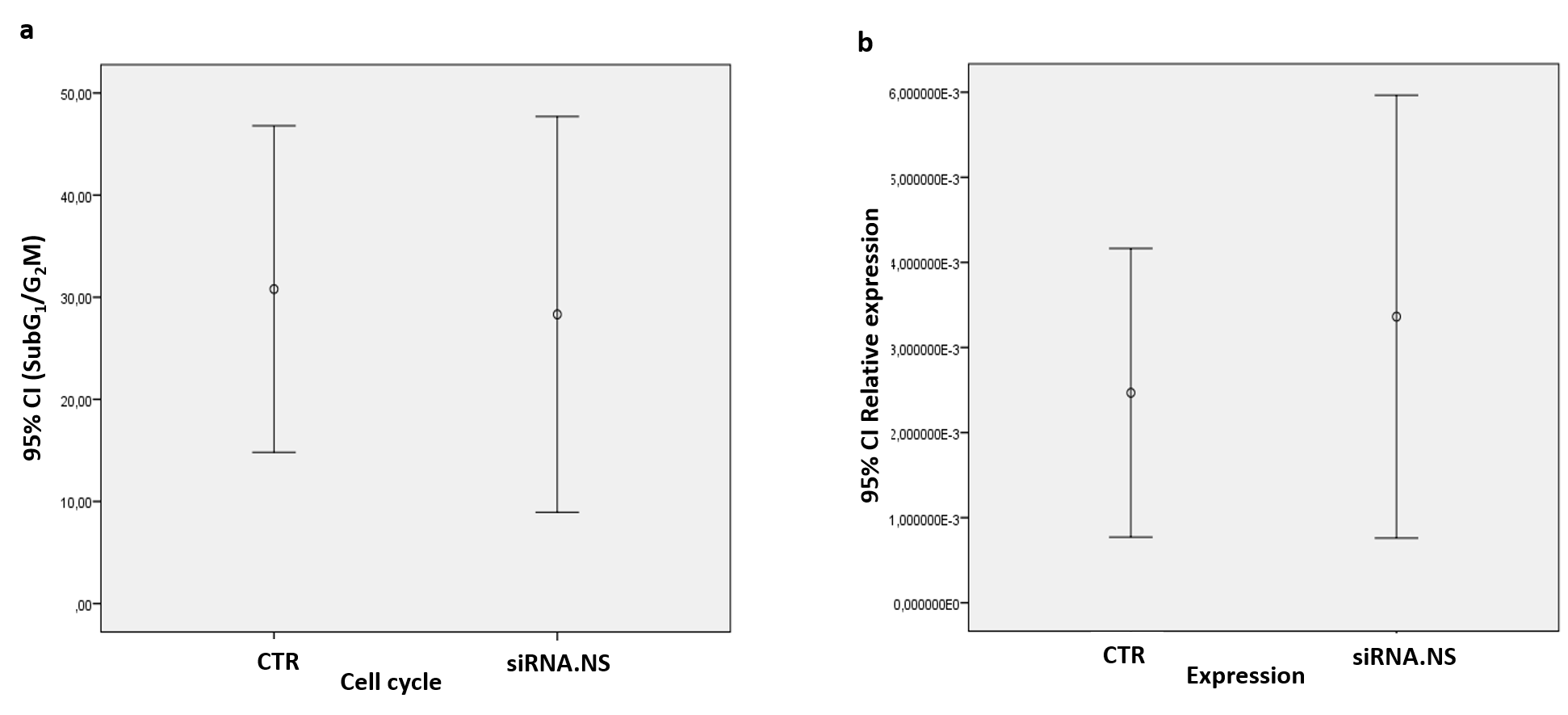

Supplement: S6 Fig — Representation of the 95% confidence interval of % of cells in the SubG1 and G2/M phases (a) and mRNA expression (b) for control (CTR) and transfected with non-specific siRNA (siRNA.NS) cells. (TIF) [file pone.0212581.s006.tif]

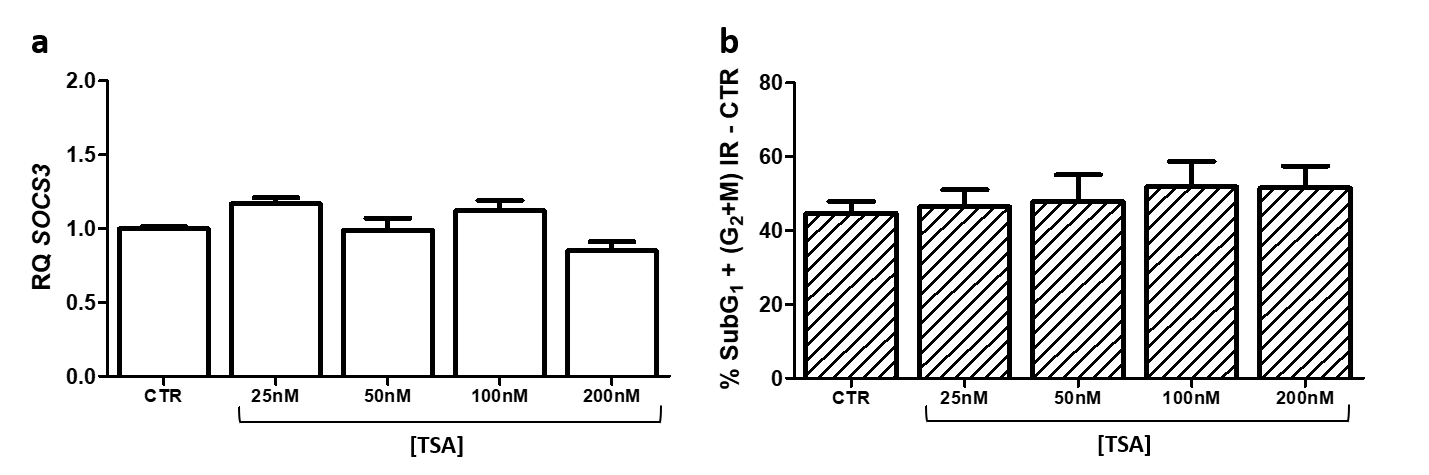

Supplement: S7 Fig — White bars show mean ± SD of SOCS3 mRNA expression in the different experiments (a). Striped bars represent mean percentages ± SD of cells in SubG1 and G2/M phases (b). (n≥3). (TIF) [file pone.0212581.s007.tif]
